# Accumulation of γ‐aminobutyric acid by *E* *nterococcus avium* 9184 in scallop solution in a two‐stage fermentation strategy

**DOI:** 10.1111/1751-7915.12301

**Published:** 2015-07-22

**Authors:** Haoyue Yang, Ronge Xing, Linfeng Hu, Song Liu, Pengcheng Li

**Affiliations:** ^1^ Institute of OceanologyChinese Academy of SciencesNo. 7, Nanhai Road, Shinan DistrictQingdaoShandong266071China; ^2^ University of the Chinese Academy of SciencesBeijing100049China

## Abstract

In this study, a new bacterial strain having a high ability to produce γ‐aminobutyric acid (GABA) was isolated from naturally fermented scallop solution and was identified as *E* *nterococcus avium*. To the best of our knowledge, this is the first study to prove that *E* *. avium* possesses glutamate decarboxylase activity. The strain was then mutagenized with UV radiation and was designated as *E* *. avium* 9184. Scallop solution was used as the culture medium to produce GABA. A two‐stage fermentation strategy was applied to accumulate GABA. In the first stage, cell growth was regulated. Optimum conditions for cell growth were pH, 6.5; temperature, 37°C; and glucose concentration, 10 g·L^−1^. This produced a maximum dry cell mass of 2.10 g·L^−1^. In the second stage, GABA formation was regulated. GABA concentration reached 3.71 g·L^−1^ at 96 h pH 6.0, 37°C and initial l‐monosodium glutamate concentration of 10 g·L^−1^. Thus, compared with traditional one‐stage fermentation, the two‐stage fermentation significantly increased GABA accumulation. These results provide preliminary data to produce GABA using *E* *. avium* and also provide a new approach to process and utilize shellfish.

## Introduction

γ‐Aminobutyric acid (GABA) has various biological properties such as anti‐anxiety, anti‐hypertension, and growth‐promoting effects (Siragusa *et al*., 2007). The development and application of GABA have gained popularity in recent times. Until now, chemical transformation and biotransformation were commonly used for producing GABA (Ger offen, 1970; Plokhov *et al*., 2000; Li and Cao, 2007). Although chemical transformation, which is more common in patent documentations, provides high yield and high purity of GABA, it requires toxic solvents that produce toxic residues. This limits its application in the food industry. Biotransformation, on the other hand, associates with benefits such as safe application in the food industry, environmental protection and energy saving (Komatsuzaki *et al*., 2005b).

In earlier studies, *Escherichia coli* was used to produce GABA via biotransformation. However, its use in the food industry is limited because of safety concerns. In recent studies, some harmless microorganisms such as lactic acid bacteria, yeast and *Aspergillus niger* have been used to prepare food‐grade GABA (Komatsuzaki *et al*., 2005b; Liu *et al*., 2007; Chao *et al*., 2011). Komatsuzaki and colleagues (2005a) screened eight strains of *Lactobacillus brevis* and found that the strain IFO 10025 could produce 10 g·L^−1^ of GABA using 5% glutamate (Glu) as the substrate. Qing and colleagues (2004) and Dongyun and colleagues (2006) isolated a high glutamate decarboxylase (GAD)‐producing strain by screening and optimizing fermentation conditions. The result showed that GABA concentration can reach 3.1 g·L^−1^. Plant enrichment is another form of biotransformation. It involves increasing GABA concentration in plants by external environmental stress. In Kenya and Sri Lanka, plant enrichment studies to increase GABA concentration have been performed using CTC black tea (Omori *et al*., 1998). Another study was performed in China to develop green tea containing a high GABA concentration (Mingxing and Dinghe, 2007). In Japan, many functional foods such as rice germ, rice bran, green tea, pumpkin, pepper, eggplant, tomato and orange containing high GABA concentrations have been cultivated by employing the plant enrichment method (Hui *et al*., 2002). However, GABA yield with plant enrichment is much lower than that with microbial biotransformation; hence, plant enrichment is not suitable for the mass production of GABA. Therefore, microbial biotransformation with new strains having better GAD activity and safety is favoured by researchers.

Scallops contain essential bioactive compounds such as carotenoids, dietary fibre, protein, essential fatty acids, vitamins and minerals. Therefore, scallops are believed to be a good candidate for producing biologically active and safe compounds. In China, the total area for scallop mariculture has reached 6.12 × 104 hm^2^, and the total yield has reached 9.355 × 10^5^ t. It indicates that scallop mariculture is flourishing (Ni, 2006). In recent times, scallops have been widely used in research because of the development of scallop mariculture and technology (Ni, 2006). Thus far, no study has described the production of GABA by microorganisms isolated from aquatic organisms. The concentration of Glu (a substrate for producing GABA) is higher in scallops than in other aquatic animals (Wang Jingxin, 1991). Therefore, we screened bacterial strains with GAD activity from scallops and fermented scallop solution to produce GABA. This may be a new method for high‐value utilization of marine shellfish. And enhancing the comprehensive utilization of scallop can reduce the pollution caused by scallop processing.

*Enterococcus avium* is a rare pathogen in humans; however, it is present as a part of the normal flora in the gastrointestinal tract of many individuals. *Enterococcus avium* is considered less virulent (Swaminathan and Ritter, 1999). Few studies have been conducted to determine the functions of *E. avium*. In this study, we isolated a strain of *E. avium* from a naturally fermented scallop solution. The isolated bacterial strain had GAD activity. Although the concentration of GABA produced by *E. avium* was lower than that produced by *E. coli* and lactobacilli, the bacterium was safer and had stronger environmental adaptability; i.e. it was resistant to acid, alkali, sodium azide and concentrated bile salts that kill almost all microbes, except enterococci (Xia *et al*., 2008; Zhang *et al*., 2013). The above characteristics make *E. avium* a potential alternative for producing GABA via biotransformation.

Previous study has shown that GABA production by microorganism includes two stages of cell growth and product formation (Huang *et al*., 2008). The two‐stage fermentation strategy is an innovative method that involves the regulation of culture medium conditions at different stages of microbial growth and metabolism. Compared with the traditional one‐stage fermentation, the two‐stage fermentation improves cell growth and guarantees an increased production of metabolites by microorganisms.

This study aimed to isolate and identify GABA‐producing microorganisms from naturally fermented scallop solution and to improve its GAD activity to produce GABA by fermenting scallop solution using the two‐stage fermentation strategy.

## Experimental procedures

### Chemicals

GABA was purchased from Sigma Reagent (purity ≥ 99%). l‐monosodium glutamate (l‐MSG) was purchased from Sinopharm Chemical Reagent.

### Culture medium

Scallop was purchased from Nanshan aquatic product market, Qingdao, Chian. MRS medium was purchased from Qingdao Rishi Biological Technology, Qingdao, Chian. GYP seed culture medium (Qingdao Rishui Biological Technology), GYP seed culture medium (g·L^−1^): glucose 10, yeast 10, peptone 5, sodium acetate anhydrous 2, MgS0_4_·7H_2_O 0.02, MnS0_4_·4H_2_O 0.001, NaCl 0.001, Fe_2_(S0_4_)_3_·7H_2_O 0.001, pH 6.8. Culture broth for the production of GABA was scallop solution. Scallop solution: fresh scallop was ground into a homogenate, added to distilled water at a ratio of 1:10 (w/v), then autoclaved at 120°C for 20 min. After cooling, 50 ml of the solution was put into a 100 ml conical flask to prepare for the next step of the experiment. One loop of slant culture (MRS) was inoculated into 50 ml of GYP medium in a 100 ml conical flask and incubated at 30°C without agitation for 24 h. The inoculum concentration of subsequent experiments was 1% (v/v).

### Isolation and screening of strains

Fresh scallop was ground into a homogenate and distilled water was added into the homogenate (1:10 w/v). The mixture stood at room temperature for 7 days, then the natural fermented scallop solution was obtained. One millilitre of liquid was diluted 10^7^ times with distilled water. The dilution (0.3 ml) was plated on MRS agar and placed at 30°C for 48 h under anaerobiosis to isolate strains. Twenty colonies that grew well on MRS agar were selected then transferred into 100 ml of GYP seed broth separately. In order to obtain a strain with high GABA‐producing ability, the stains in seed broth were transferred into the GYP broth containing 1% l‐MSG and were cultivated at 37°C for 48 h. The quantitative results of GABA in the fermentation liquid were analysed. Then the selected strain with high GABA‐producing ability was mutagenized with UV treatment, irradiation time 10 s, irradiation distance 30 cm and UV light wavelength 254 nm. The 16S rDNA sequence of the selected strain was detected by TaKaRa Biotechnology (Dalian, Dalian City, Liaoning Province, China). The sequencing result was input into NCBI to identify the strain.

### Scallop solution fermented by selected strain in two‐stage control strategy

In the fermentation process, the effects of initial carbon source (glucose concentration) addition, pH and temperature on cell growth and GABA production were investigated. Scallop solution and a substrate feeding fermentation with the two‐stage control strategy were applied to obtain a more efficient, simpler and cheaper GABA production.

### Analytical procedures

GABA and l‐MSG in the scallop solution were quantitatively analysed by high‐performance liquid chromatography. Chromatographic column was C18ODS reverse‐phase column (250 mm × 4.6 mm, 5 μm). Mobile phase including 0.1 mol·L^−1^ of phosphate buffer (pH 6.0) 52%, methanol 46%, tetrahydrofuran 2%, was filtered with 0.22 μm of membrane filter and deaerated by ultrasonic treatment. The flow rate was 1 ml·min^−1^, and the detection wavelength was 338 nm. The derivative agent was 10 ml of o‐phthalaldehyde, which was dissolved by 0.5 ml of methanol, 2 ml of 0.2 mol·L^−1^ borate buffer solution (pH 10.0) and 30 μl of 2‐mercaptoethanol. One hundred microlitres of each sample was filtered with 0.45 μm of membrane filter, then mixed with 100 μm of derivative agent for 90 s. Finally, 20 μl of mixed liquor was detected. GABA standard curve was made by external standard method and the concentration of GABA was calculated. Cell growth was monitored by measuring dry cell mass.

### Statistical analysis

All experiments were performed in triplicate. Data are presented as mean ± SD. Statistical significance was determined by Student's *t*‐test. Values with *P* < 0.05 were considered significant.

## Results

### Screening and identification of GABA‐producing strain

In total, six out of 20 cultured strains produced GABA (Table 1); from these, strain A13 showed the highest production of GABA (2.20 g·L^−1^). Next, strain A13 was mutagenized using UV radiation. Single factor experiment and response surface analysis (data not shown) showed that GABA reached a highest concentration of 3.17 g·L^−1^ (after culturing for 72 h).

**Table 1:** The high‐performance liquid chromatography method for the determination of GABA‐producing strains

Strain no.	A1	A6	A7	A11	A12	A13
GABA (g·L^−1^)	1.90 ± 0.14	1.97 ± 0.13	2.07 ± 0.09	1.52 ± 0.17	1.84 ± 0.06	2.20 ± 0.18

To confirm the A13 strain, its 16S rDNA sequence was amplified and 1400 bp of the DNA sequence was determined (data not shown) by TaKaRa Biotechnology(Dalian). The GenBank database was used to search for genes having similar 16S rDNA sequences. This revealed that the A13 strain had a 99% sequence identity with *E. avium* (GenBank accession No. NBRC 100477 and GenBank accession No. ATCC 14025). Based on these results, the A13 strain was considered to be an *E. avium* strain and was designated as *E. avium* 9184. The strain was deposited at the China General Microbiological Culture Collection Center (CGMCC 9184).

### Time evolution of GABA production in unoptimized scallop solution

**Figure 1:**
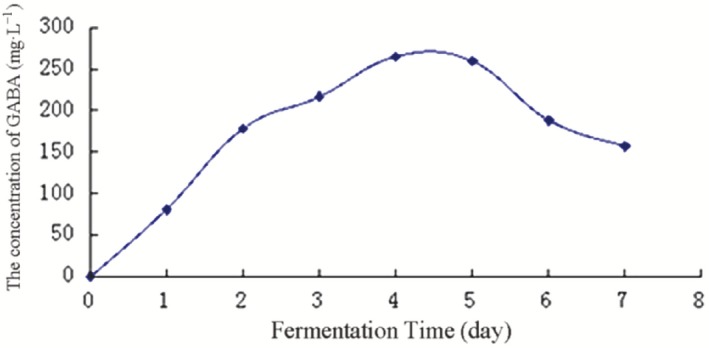
Time course of GABA concentration in unoptimized scallop solution.

**Figure 2:**
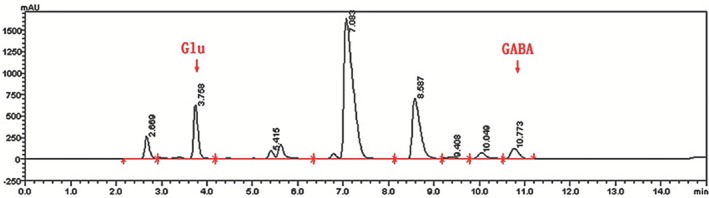
High‐performance liquid chromatography of unoptimized scallop solution fermented with *E* *. avium 9184* (24 h fermantation).

**Figure 3:**
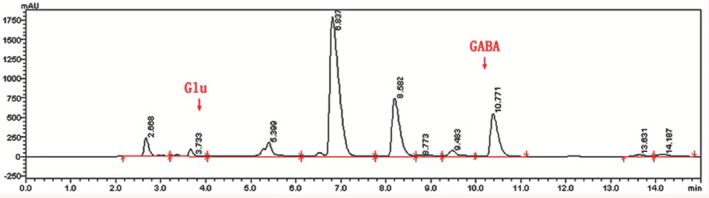
High‐performance liquid chromatography of unoptimized scallop solution fermented with *E* *. avium 9184* (72 h fermentation).

After fermentation for 1 day at 37°C, the concentration of GABA was 71.29 mg·L^−1^ and reached a maximum of 265.22 mg·L^−1^ on the fourth day of fermentation (Fig. 1). The concentration of Glu in dry scallop is approximately 7.99 g/100 g (Wang Jingxin, 1991). The concentration of Glu decreased and that of GABA increased gradually with fermentation time (Figs 2 and 3), indicating that *E. avium* 9184 used nutrients present in the scallop solution to convert Glu into GABA.

### *E* *. avium* 9184 fermented scallop solution in the two‐stage fermentation strategy

A previous study has shown that GABA production by microorganism involves two stages, i.e. cell growth and product formation (Huang *et al*., 2008). Therefore, the two‐stage strategy involving a control of culture conditions at different stages was established and used for enhancing GABA production in scallop solution.

#### The first stage of the two‐stage fermentation strategy: effect of temperature, pH and glucose concentration on cell growth

High initial concentration of l‐MSG restricts cell growth. Moreover, the concentration of Glu in scallops is relatively high. Therefore, in the first stage, l‐MSG was not added and only cell growth‐promoting factors were added to obtain maximum biomass. The addition of cell growth‐promoting factors give microorganisms the opportunity to produce more GAD.

**Figure 4:**
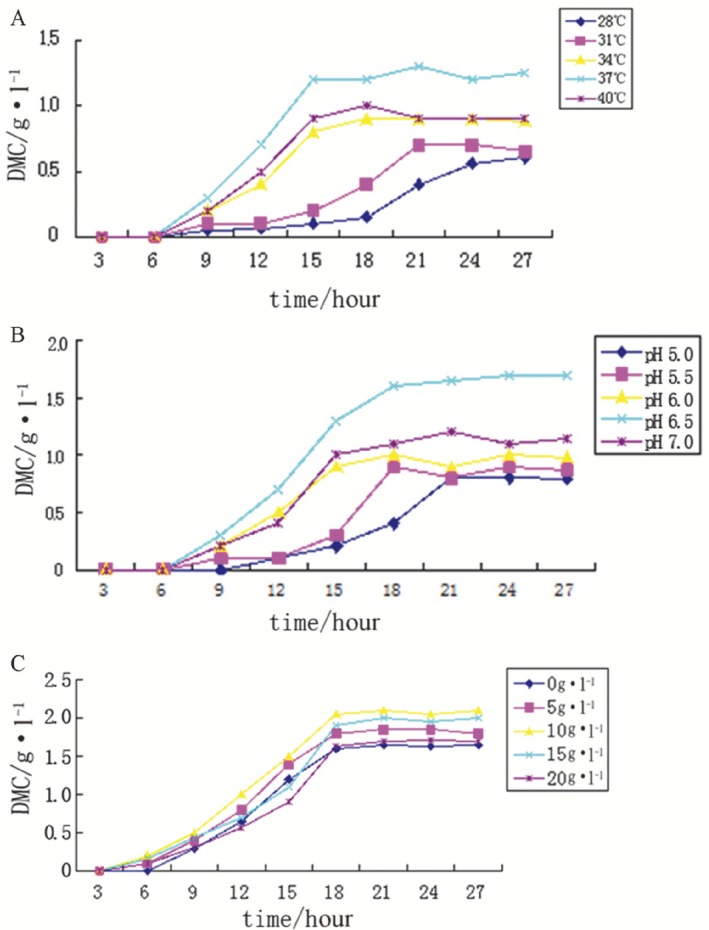
The first stage of the two‐stage fermentation: effect of pH, temperature and glucose addition on cell growth. A. pH 7.0 and various temperatures. B. 37°C and various pH values. C. pH 6.5, 37°C and various glucose concentrations.

To investigate the effect of temperature on the growth of *E. avium* 9184 in the scallop solution, the cultures were maintained at 28°C, 31°C, 34°C, 37°C, and 40°C (Fig. 4A). The highest cell biomass was obtained at 37°C, and the cells reached the stationary phase after 15 h of cultivation.

To investigate the effect of pH on the growth of *E. avium* 9184 in the scallop solution, the pH of the cultures was controlled at 5.0, 5.5, 6.0, 6.5, and 7.0 (Fig. 4B). The highest cell biomass was obtained at a pH of 6.5, and the cells reached the stationary phase after 18 h of cultivation. Cell growth was strongly inhibited at a pH of 5.0.

Carbon resource (glucose) is one of the well‐documented factors influencing cell growth during fermentation. Our results showed a considerable variation in the yield of cell biomass at different glucose concentrations (Fig. 4C). The highest cell biomass (2.10 g·L^−1^) was obtained when the glucose concentration was 10 g·L^−1^, and the cells reached the stationary phase after 21 h of cultivation.

#### The second stage of the two‐stage fermentation strategy: effect of pH, temperature and L‐MSG concentration on GABA production

Our results indicated that the optimum initial pH for GABA production was 6.0 (Table 2) and that the optimum temperature was 37°C (Table 3), which was also the most suitable temperature for cell growth. GABA accumulation reached a maximum value after 96 h of cultivation. The addition of 10 g·L^−1^ of l‐MSG provided the highest concentration of GABA (3.71 g·L^−1^) after 72 h of cultivation (Table 4); this concentration was 3.17 g·L^−1^ with traditional one‐stage fermentation.

**Table 2:** The second stage of the two‐stage fermentation: effect of pH on GABA (mg·L^−1^) production

Day	37°C, initial concentration of l‐MSG 405 mg·L^−1^ and various pH values				
	5.0	5.5	6.0	6.5	7.0
1	56.2 ± 8	66.7 ± 9	90.3 ± 8	84.9 ± 10	70.2 ± 6
2	176.4 ± 17	184.3 ± 13	207.2 ± 12	173.6 ± 21	168.7 ± 23
3	213.1 ± 19	266.4 ± 17	303.7 ± 26	254.7 ± 27	226.2 ± 25
4	191.5 ± 17	239 ± 22	286.4 ± 21	239.4 ± 19	212.6 ± 33

**Table 3:** The second stage of the two‐stage fermentation: effect of temperature (°C) on GABA (mg·L^−1^) production

Day	Initial pH 6.0, initial concentration of l‐MSG 410 mg·L^−1^ and various temperatures				
	31	34	37	40	43
1	51.0 ± 7	64.2 ± 11	92.9 ± 12	88.9 ± 10	90.6 ± 10
2	176.2 ± 13	181.1 ± 11	208.2 ± 9	191.0 ± 14	192.3 ± 16
3	201.7 ± 29	219.9 ± 16	306.1 ± 16	295.7 ± 17	286.2 ± 23
4	165.5 ± 14	179 ± 20	284.5 ± 21	269.8 ± 18	263.2 ± 19

**Table 4:** The second stage of the two‐stage fermentation: effect of initial L‐MSG concentration (g·L^−1^) on GABA (mg·L^−1^) production

Day	37°C, initial pH 6.0 and various initial added l‐MSG concentrations (g·L^−1^)				
	0	5.0	10.0	15.0	20.0
1	81.9 ± 8	315.6 ± 46	920.3 ± 8	874.9 ± 103	570.3 ± 67
2	197.3 ± 21	984.3 ± 183	2706.2 ± 12	1973.4 ± 221	1618.7 ± 123
3	311.3 ± 39	1743.1 ± 169	3713.7 ± 226	3254.7 ± 297	2677.2 ± 295
4	271.5 ± 31	1539 ± 122	3586.4 ± 217	2939.2 ± 199	2312.6 ± 263

## Discussion

GAD activity of microorganisms is important for producing GABA. In prokaryotic microorganisms, it is believed that GAD is associated with acid‐proof mechanism of cell growth (Christensen *et al*., 1999; De Biase *et al*., 1999; Ueno, 2000). The acid‐proof system mainly works through a synergistic reaction between GAD and a relevant Glu/GABA reverse transporter. The reverse transporter transports exogenous Glu into cells, where it is irreversibly decarboxylized by GAD to form GABA. GAD can only be induced in an acidic environment of high permeability or low permeability, and low oxygen and is expressed under the molecular cascade control of H‐NS and RpoS factors (De Biase *et al*., 1999).

To produce GABA, *E. avium* requires proteins, carbon resource (glucose), Glu/L‐MSG, trace elements, etc. Scallops contain 20% protein, 0.5% reducing sugar and various trace elements that can be used as nutrients for producing GABA. GABA concentration in the fermentation medium can be increased by optimizing the fermentation conditions; i.e. by adding carbon source (glucose) and fermentation substrate (l‐MSG) and by adjusting the pH and temperature of the fermentation medium.

Two‐stage pH and temperature control of substrate feeding fermentation: Substrate feeding and pH control are ideal tools for optimizing the fermentation process. The substrate feeding method is generally applied to overcome substrate inhibition during cell growth (Weuster‐Botz *et al*., 2001; He *et al*., 2009). To avoid the inhibition of cell growth at higher l‐MSG concentrations, a two‐stage process involving pH and temperature control with substrate feeding strategy was developed to produce GABA by *E. avium* 9184.

### Glucose

The addition of moderate concentrations of glucose to scallop solution can make up for the lack of carbohydrates, boost the growth and breeding of cells (Jones *et al*., 1999), and quicken the fermentation process. In addition, it can slightly increase extracellular pressure, which induces the expression of GAD. *Enterococcus avium* cells may shrink due to high osmotic pressure if a large amount of glucose is present in the scallop solution, which may inhibit its growth and reproduction (Nguyen Thi Minh *et al*., 2008). In this study, a glucose concentration of > 15 g·L^−1^ inhibited cell growth and reproduction because of high osmotic pressure, thus resulting in decreased GABA production. When glucose concentration was less then 10 g·L^−1^, enough carbon source was not available for the growth of *E. avium*, which in turn may have decreased GAD expression.

### Glu/L‐MSG

Glutamate/L‐MSG is the optimum substrate for GAD, which catalyses the reaction of Glu with H^+^ to produce GABA (Chunlong *et al*., 2013). In some special cases, GABA can also be produced from ornithine or butanediamine. However, all these substances are derived from Glu; therefore, Glu can be regarded as the only source of GABA (Xiaomin and Xianrong, 1994). A study showed that 100 g of dry scallops contains 7.99 g of Glu (Wang Jingxin, 1991), which is far more than that present in other organisms. However, experimental data showed that the concentration of Glu in the scallop solution could not meet the Glu requirement of *E. avium* 9184. Addition of appropriate concentrations of l‐MSG (approximately 0–15 g·L^−1^) can improve the yield of GABA. However, high concentration of l‐MSG can directly induce toxic effects such as apoptosis (Rudolf *et al*., 2013); moreover, reduction in the pH (Glu is an acidic amino acid) of the scallop solution can indirectly affect the metabolism of *E. avium*. After optimization in the second stage, addition of 10 g·L^−1^ of l‐MSG to the scallop solution minimized the toxic effects on cells and met the Glu requirement of *E. avium* 9184.

### pH

Usually, the optimum pH for GAD activity is 3.8–5.0 and mainly 4.0–5.0 (Shengyuan *et al*., 2007). When pH is > 6.0, GAD is easily dissociated (Jianjun, 2004). Therefore, appropriate reduction in the initial pH of the medium can not only induce the expression of *GAD* and increase the activity and stability of GAD but can also supply H^+^ required for GABA production. However, by changing membrane charge and permeability, the pH will affect the absorption of nutrients by and intracellular metabolic activity in the bacteria (Casanie *et al*., 1999). Reduction in the pH of the scallop solution to a level that exceeded the ability of *E. avium* to maintain a dynamic intracellular H^+^ balance inhibited the growth and metabolic activity of the bacteria and even resulted in bacterial death (Zaitseva *et al*., 2004). Therefore, a high or low pH is not conducive for producing GABA. Proper acidification of the culture medium favoured the conversion of Glu to GABA; however, very low pH inhibited the growth and metabolic activity of *E. avium*. If the pH was very high (i.e. alkaline), GABA was converted to succinic acid semialdehyde by GABA transaminase, which was not conducive for GABA production. In this study, the initial pH of the scallop solution was set to 6.5 to create a suitable environment for the normal growth of *E. avium*; the pH was then set to 6.0 to promote the conversion of Glu to GABA.

### Temperature

GABA is the secondary metabolite of many microorganisms. Gassm and colleagues (1997) suggested that lower temperature prolonged the stability of microorganisms, which helped increase the yield of secondary metabolites. However, the optimum reaction temperature of microbial GAD is relatively higher, mainly between 30°C and 50°C (Shengyuan *et al*., 2007). Therefore, in this study, the fermentation temperature was maintained at 37°C, which guaranteed good growth of *E. avium* 9184 and good activity of GAD.

### Fermentation time

Along with the production of GABA, H^+^ ions were consumed and pH increased to a maximum of 10.0 in the scallop solution. This resulted in the decomposition of GAD, which inhibited GABA production. In an alkaline environment, the synthesized GABA is catalysed by 7‐aminobutyrate transaminase, which transfers α‐ketoglutaric acid‐forming succinic semialdehyde and Glu. The SSA is then oxidized to form succinate that enters the Krebs cycle and is finally decomposed to water and carbon dioxide (Satya Narayan and Nair, 1989). Therefore, fermentation time should be strictly controlled to ensure that GABA concentration in the scallop solution reaches a maximum value. Experimental data showed that GABA concentration in the scallop solution reached a maximum value after 96 h of fermentation using the two‐stage strategy.

## Conclusion

In this study, scallops were used as a new raw material for producing GABA; *E. avium* 9184, which has high GABA‐producing ability, was used as the fermentation strain, and the fermentation process was optimized. A two‐stage pH and temperature control with substrate feeding fermentation process was developed for GABA production. After 21 h of cultivation at 37°C, pH 6.5 and initial glucose concentration of 10 g·L^−1^ (to ensure higher cell biomass), pH was adjusted to 6.0, temperature was maintained at 37°C, and 10 g·L^−1^ l‐MSG was added to the culture medium to achieve optimal GABA production. Using this strategy, GABA accumulation increased markedly and reached 3.71 g·L^−1^ at 96 h compared to 3.17 g·L^−1^ using the optimized traditional one‐stage fermentation process. *Enterococcus avium* used scallop solution as the fermentation medium, which reduced the production cost of GABA and simplified the process of culture preparation (trace elements were not required). Besides GABA, scallop fermentation medium also contains other amino acids such as glycine, arginine, alanine and lysine. After separation and purification, these amino acids can be used as flavouring agents or drug additives, thus adding to the economic value. The two‐stage fermentation strategy can be used to improve GABA accumulation in scallop solution. And drawing lessons from this study, other low‐value marine products or residues may also achieve their high utilization.

## Conflict of Interest

None declared.
